# Long Term Exercise Engagement of Adults Living With Type Two Diabetes Is Enhanced by a Person-Centred Care Approach Delivered by Knowledgeable, Well Trained Health Care Professionals

**DOI:** 10.3389/fresc.2021.692311

**Published:** 2021-07-14

**Authors:** Leigh Hale, Christopher Higgs, Donna Keen, Catherine Smith

**Affiliations:** Centre for Health, Activity, and Rehabilitation Research, School of Physiotherapy, University of Otago, Dunedin, New Zealand

**Keywords:** type 2 diabetes, self-management, adults, self-efficacy, person-centred care, exercise, education

## Abstract

**Background:** Regular engagement in exercise or physical activity is a key evidence-based recommendation in the self-management of type 2 diabetes (T2D). The Diabetes Community Exercise Programme (DCEP) is an exercise and educational programme aimed at supporting adults living with T2D to take control of their health and to live well with T2D. It was specifically developed to enhance the self-efficacy of people to engage in exercise for a long term and is underpinned by the spirit of motivational interviewing. This study explores what DCEP attendees and health care professionals (HCPs) who deliver the programme perceived DCEP to be and what motivated attendance. Such insights further the knowledge of how people with T2D can be supported to engage in exercise or physical activity programmes.

**Method:** This qualitative study used open-ended interviews of 17 DCEP attendees and 12 HCPs delivering DCEP. Interviews occurred at the completion of the initial twice-a-week, 12-week duration part of the programme and prior to attendees starting with a twice-weekly maintenance exercise class, which forms the second part of the programme. Interviews were audio-recorded, transcribed verbatim and analysed with the General Inductive Approach.

**Results:** The two themes constructed from the analysis were *person-centred care* and *attention to logistics and administration*. Person-centred care comprised four subthemes: monitoring, individualised exercise within a sociable group setting, flexible education and discussion, and HCP training, and these components appeared to support attendees to engage in exercise. The second theme spoke about the processes, that was either present or that should be included, that enabled DCEP delivery, such as appropriate venues, flexible approaches to time of day and the requirement of good administrative support.

**Conclusion:** The Diabetes Community Exercise Programme did motivate people with T2D to engage in exercise. Important to this was the emphasis on a person-centred approach that focussed on the health status monitoring and educational and social aspects of the programme, which in turn facilitated exercise engagement. Knowledgeable HCPs who require training in the delivery of person-centred care to tailor the exercise and education to the individual is imperative. Equally important are optimal exercise environments and well-trained administrative support.

## Introduction

Type 2 diabetes (T2D) is a substantial and increasing health problem worldwide including in New Zealand (NZ), where over 25,000 people are estimated to have T2D (1). Especially concerning in NZ is the high prevalence of T2D among Māori and Pacific people, and people living in low socioeconomic areas; people who find accessing appropriate, quality health care challenging (1). The current NZ Ministry of Health quality standards for diabetes care include basic care, self-management and education, all of which should be culturally relevant and tailored to individual needs (2). Self-management support is internationally a key component of frameworks detailing the management of long-term conditions, such as T2D (3). Evidence verifies that supporting self-management of health can improve the short-term glycaemic control in people with T2D, including in culturally diverse populations (4). Further, such support should be tailored for the individual and their community, ensuring that approaches used are culturally, socially, and demographically appropriate to enable sustainability and accessibility (4).

Regular engagement in exercise or physical activity is a key evidence-based recommendation in the self-management of T2D (5–9), at least for exercise programmes of short duration. For example, a network meta-analysis investigating different exercise training modalities demonstrated that a combined programme of supervised aerobic and resistance exercise improved glycaemic control, but none of the included 37 studies had an exercise programme duration range of longer than 6 months (10). Additionally, while the improvement of HbA1c using self-management approaches, including exercise, was shown at 6 months post-intervention, by the 12- and 24-month follow-up, these improvements had reduced (4, 11). This latter finding is not surprising given that T2D is a long-term condition, and so lifestyle changes, including engagement in physical activity, will only positively impact glycaemic control while being undertaken. In other words, for exercise to be beneficial in glycaemic control, the person with T2D needs to engage in it regularly for life.

Engaging long term in a lifestyle behaviour, such as exercise or physical activity, is challenging for anyone, including those living with T2D (12). While a diagnosis of T2D in itself can be an initial internal motivator for a lifestyle change, maintaining internal motivation is complex and often slow to develop, and can cause internal conflict, frustration, and a need for continual external prompting and ongoing support (13). Studies exploring facilitators and challenges to physical activity engagement in people with T2D identify many generic factors such as time, training tools and facilities, factors which then become further nuanced when exploring cultural, social and gender influences (12, 14).

In response to the increasing prevalence of T2D in our region of NZ, we developed the diabetes community exercise programme (DCEP) with a focus on working with Māori and Pacific people and those of lower socioeconomic status. The overarching aim of DCEP is to support adults living with T2D to take control of their health and to live well with their long-term condition. To achieve this aim, the emphasis is on exercise self-management and exercise self-efficacy to engage people with T2D into exercise for a long term. DCEP thus comprises both exercise and educational components, tailored to individual needs. An initial twice-a-week 12-week programme of exercise and education is followed up by attendance two times a week at a maintenance exercise class. The programme has been described in detail in a previous study (15–17). DCEP is underpinned by the spirit or “heart-set” of motivational interviewing, encompassing partnership, acceptance, compassion and evocation (18). Addressing known barriers to exercise, DCEP was developed based on values of partnership, acceptance, compassion and person-centred goal-setting; safety (prescribed and monitored by a physiotherapist and a diabetes nurse); easy access (community-based); being culturally appropriate (informed by extensive consultation, including whānau/family) and being freely available. Such an approach is not currently the “usual” diabetes care in NZ. DCEP has been running for 11 years, and its effectiveness is presently under investigation in a randomised controlled trial (RCT). This trial is currently being conducted to test the effectiveness of the programme on glycated haemoglobin (HbA1c), which aims to allocate 220 adults (age ≥35 years) with a diagnosis of T2D to either DCEP or usual care [which includes appropriate medication, advice regarding diet and physical activity participation and referral to the DESMOND programme (a 1-day education programme designed to support people living with T2D)] (19). The trial data are presently being analysed (15).

DCEP attendees over the years speak highly of the classes, and indeed, 12% of referrals are “word-of-mouth” from a DCEP attendee (16, 17). Many participants (about 39%, pre-RCT data analysed in 2018) go on to attend the maintenance group exercise programme (20), and there is an “End of Year” social event, which ~70 participants attend. The acceptability of DCEP has been established by the high retention rate (70% over the initial 12-week programme) and *via* qualitative interviews (16, 17).

In our current community-based RCT, DCEP was provided in two cities in community exercise venues. Both cities are small. City 1 has a current population of 114,347 people [Pākehā (non-Māori) 87%, Māori, 9%, Pacific Island 3%]. City 2 has a current population of 57,100 [Pākehā (non-Māori) 88%, Māori, 14%, Pacific peoples 3%]. While DCEP is underpinned by a specific philosophy and key principles (as described earlier), it was developed in City 1. Therefore, context-specific modifications for implementation in new centres to ensure appropriate delivery and successful uptake (for example local needs and cultures, and idiosyncrasies of local health systems and processes) may be required. To this end, the study was introduced into our new centres sequentially, identifying the key processes and subtle changes required for implementation into a new site. Based on our 11 years of knowledge, we first introduced the study into a new site into City 1 and then later into City 2. Over the 1-year course of the trial, we had 6.8% (8/85) attrition from the 12-week initial programme in the intervention arm, with the mean (median, range) attendance rate of 11 (10, 1–24) and 14 (19, 0–24) per session in Cities 1 and 2, respectively. This trial thus gave us the opportunity to explore, with attendees, randomised to the intervention arm, and health care professionals (HCPs) and providers involved in delivering DCEP in the two sites, what they considered the essence of DCEP to be and what makes people attend (and continue attending) the initial 12-week programme, or what does not.

This qualitative study aimed to explore, at the end of the initial 12-week intervention, what attendees and HCPs who deliver the programme perceived DCEP to be and what motivated attendance and whether DCEP is addressing its purpose of facilitating exercise self-management and exercise self-efficacy. These insights would further the knowledge of what enables people with T2D living in NZ, and elsewhere, to engage in exercise programmes.

## Methods

### Design

As we were exploring experiences of a health service, we chose the qualitative evaluative methodology used for assessing health programmes and services (21). We collected data using semi-structured interviews or focus groups depending on participant preference. Data were analysed with the General Inductive Approach, a pragmatic approach specifically for the analysis of evaluation data (22). The study was approved by the NZ Health and Disability Ethics Committee (17/CEN/241). The RCT is registered with the Australian New Zealand Clinical Trials Registry (ANZCTR): ACTRN12617001624370p. This report adheres to the COnsolidated criteria for REporting Qualitative research (COREQ) checklist (23).

### Recruitment and Eligibility

Participants for this qualitative study were recruited from those randomised to the intervention arm of our RCT (attendees) and from all HCPs involved in the delivery of DCEP. Eligibility and recruitment for our RCT have been described in detail elsewhere (15), but briefly, people diagnosed with T2D aged >35 years, who were randomised to the intervention arm and who were cleared by their GP to participate in exercise, were eligible. Attendees were recruited into the trial *via* health care providers or *via* public media advertising.

### Data Collection

Interviews and focus groups were conducted by one of three female interviewers, all trained in, and with previous experience in, qualitative methodology. Interviewers were research assistants with bachelor's degrees who came from a variety of backgrounds (nursing, psychology, social science) and ethnicities [Māori, Pākehā (non-Māori)]. These interviewers were known to all participants prior to the interviews occurring *via* the organisational aspects of the RCT. DCEP attendees were only interviewed. Due to logistical or availability reasons, data from HCPs were collected either by interview or by focus group. Interviews took place at mutually agreeable venues (homes or clinic), were audio-recorded and took on average 1 h. Field notes were made by interviewers immediately following data collection, notes that were later used to assist the analysis. No non-participants attended the interviews although attendees were able to bring a support person if so desired. All interviews were transcribed verbatim by a commercial transcribing firm. No repeat interviews were required. The wider research team along with the interviewers developed the interview guide (see Table 1).

**Table 1:** Interview guide.

**Diabetes Community Exercise Programme attendee questions**
Describe for me your experience of going through the DCEP programme from start to finish?What could be better/different?How valuable you think DCEP is to your community?How well you think DCEP has been adapted to your local community's needs?What worked really well?How well you think DCEP has been accepted to your community?How well DCEP has met the needs of those who have attended?Your views on how likely DCEP is to remain in your community in the future?How has it changed your motivation to exercise?
**Health care provider questions**
What do you think about DCEP? Why?What are the important components of DCEP to you? Why?If you could change something about DCEP, what would it be? Why?What was it like running DCEP?What went well?What didn't?What did you learn?How did it influence your practise?

### Data Analysis

The General Inductive Approach guided analysis (22). Two researchers (CS and LH) began by listening to the DCEP participant recordings and made notes about participant reflections on the DCEP programme in a notebook and compared notes. Then, one researcher (CS) read and coded participant transcripts and made notes about the possible lower-order categories. As CS progressed through transcripts, she identified the higher-order categories and recorded them in an excel spreadsheet. As higher-order categories were agreed upon by the research team discussion, CS began to code the HCP interview and focus group transcripts. As more higher-order categories were added, further research team discussions lead to the construction of themes and the transference of higher-order categories into new spreadsheet pages. Participant quotes that supported each higher-order category were added in a column alongside, and participant details were attributed to each quote.

### Trustworthiness

Illustrative models of the analysis were developed at each stage, and these, along with lower-order categories, higher-order categories and developing themes, were shared with all members of the research team on a weekly basis. The research team sent feedback and suggestions, and theme development was discussed by the team at a fortnightly meeting. The final analysis was presented and verified at workshops held in both cities attended by DCEP attendees and HCPs.

## Findings

A total of 17 DCEP attendees consented to participate, of whom seven were from City 1. The group included six men and 11 women, ranging in age from 39 to 76 years. Table 2 reports the demographic details of all 15 DCEP attendees. Twelve (female = 10) HCPs were interviewed, which included two physiotherapists, four nurses, a programme developer, a pharmacist, a podiatrist, a dietitian, an administrator and a counsellor. Data saturation was considered reached; thus, no further interviews were required.

**Table 2:** Demographic details of participants.

**Participant**	High/Low* Attendance at DCEP	**Age years**	**Sex**	Ethnicity**
**City 1**
P 840	Low	39	M	NZE
P529	High	73	F	Cook Island Māori
P686	Low	54	F	NZE
P519	Low	55	F	NZE
P542	High	73	M	Māori
P917	Low	72	M	NZE
P595	High	76	M	NZE
**City 2**
P205	High	72	M	NZE
P238	High	41	F	NZE
P280	High	70	F	NZE
P324	High	63	F	NZE
P373	High	70	M	NZE
P639	High	54	F	Māori
P887	High	51	F	NZE/Māori
P920	High	56	F	NZE
P175	Low	53	F	NZE
P933	High	64	F	Māori

*DCEP, Diabetes Community Exercise Programme; NZE, New Zealand European*.

*High attendance was defined as attending ≥50% of all available classes.

**As per New Zealand Census (New Zealand European, Māori, Samoan, Cook Island Māori, Tongan, Niuean, Chinese, India, Other), each ethnic group includes all those who have identified with it, so people may be counted in more than one group.

We identified two themes important to what attendees and HCPs perceived DCEP to be and what appeared to motivate attendance, and these were: (1) *person-centred care*, a major theme that comprised four subthemes, and (2) *attention to logistics and administration*, a minor theme with no subthemes. We detail these themes and subthemes below, illustrating them with quotes attributed in the following way: DCEP attendees are attributed with their unique study number, e.g., P238; HCPs are attributed with their profession, location (either C1 for City 1 or C2 for City 2), and where more than one of each profession in an area, a numerical 1 and 2 (e.g., Nurse,C2,2).

### Theme 1: Person-Centred Care

That DCEP revolved around person-centred care was a major theme overt in the data. Within this major theme were four subthemes, namely, *monitoring, individualised exercise within a sociable group setting, flexible education and discussion* and *HCP training*. In each subtheme, person-centred care was evident. We discuss each subtheme in turn below.

#### Monitoring

Monitoring of attendee health status was undertaken by both the nurse and the physiotherapist prior to and during all exercise sessions. While primarily done for safety concerns, monitoring was done in such a person-centred manner that it became a motivator for attendance. It was done both obviously and indirectly. At the start of a session, the nurses monitored and recorded in their personal notebooks blood sugar levels (BSLs) and blood pressure (BP) of attendees while simultaneously checking for any problems or health issues by indirect questioning and discussion: “*regularly come and get their blood pressure and blood sugar checked each time which they don't really need to but it is a chance for some one-on-one you know, just slightly away from the group*” (Nurse,C1,1).

Additionally, the physiotherapist and nurse monitored attendees during the exercises, watching for and correcting unsafe exercise techniques or for signs of physiological stress. Attendees were aware they were being unobtrusively monitored and consequently felt safe participating: “*It made you feel comfortable and safe. If they weren't, if, they, you didn't have the nurse or the um, physio there, I don't know if I would have done the course”* (P238).

Documented positive changes in BSLs and BP facilitated the motivation of attendees to continue involvement: “*On the 24th, my blood pressure was 160 over 90 which is quite high.And then, as we keep going down, it went down to 140 over 90.And then it went to 132 over 82. Um and then towards the end, like I was getting 124 over 82”* (P238). Some HCPs considered this frequent BP and BSLs monitoring unnecessary and reinforced attendee dependence on HCPs: “*They don't know that they don't need it. It's sort of created this whole monster, really, of a line-up for blood pressures”* (Nurse,C2,1). Furthermore, for some DCEP attendees, it appeared they only came to DCEP to have their BSLs and BP monitored. These HCPs did acknowledge, however, that this obvious monitoring created an opportunity for more discreet (and probably important) monitoring “*How're you doing, how's it going and how are you finding it?*” (Nurse,C1,1).

In monitoring the exercises, the physiotherapists sometimes expressed concern about balancing safety with motivation when less-than-perfect exercise techniques were observed: “*I'm more about them doing something that they enjoy and if that's the way they enjoy it.I'd rather they did something than kinda go” “Oh she's always really picky about how I do that, I don't wanna do that one anymore.”* and instead asking …*. “Sure that's not sore?"* (PT,C1).

#### Individualised Exercise Within a Sociable Group Setting

DCEP was primarily set up as an exercise programme focussing on exercise prescription to address T2D. The emphasis, however, on person-centred care meant that the physiotherapists tailored the exercises not only for T2D but for individual attendee goals or needs. Although the exercise was tailored to the person, it took place in a group setting, thereby emphasising the social aspect, and this social context, in turn, promoted the focus on attendees: “*I think by the end of the programme to, one of the biggest benefits was actually um, the camaraderie that you had with everybody. Um, all you know, hi XXX [name] how are you today and you were the same, kind of got to know people a bit and thoroughly enjoyed that. And that probably was um, took me going back, XXXX [name] and I kept us going back on the maintenance programme*” (P324). The social aspect, as opposed to the exercise, appeared to motivate attendance: “*Do I enjoy it here? I love it. Are the people great? Fantastic. And the group is fantastic, we've made new friends um, and they're all respective of one another, but they all have tears and laugh and joke amongst themselves*” (P205). The social atmosphere assisted people to exercise: “*Like, we have a laugh, and we sit on the bikes going flat out, talking away*” (P280).

Many attendees expressed a preference for DCEP over attendance at a regular gym, where costs or appearance could be prohibitive: “*It's like, when people go to the gym, they've got all the flash gears on, you know, just to be looked at*” (P933). Attendees also valued the relationships provided by DCEP: “*Because it's a group situation. Whereas you go to the gym, you are an individual dropped in and nobody talks to you”* (P280).

The individualised nature of the exercise prescription (as opposed to a standard set of exercises for all) appeared to further contribute to a motivation to attend, as, for the attendees, exercises needed to be meaningful and contribute toward balancing their needs. Attendees had different priorities, often not directly related to their T2D, for example, recent surgery or awaiting surgery, and were delighted that the exercises could be tailored to their needs: “*I am 13 months out of a triple bypass so I have a few issues with my chest. She* [physiotherapist] *has been working with me to exercise and strengthen chest muscles, etc. This is something that would never have happened if I hadn't had been involved in this*” (P373). For others, exercise needed tailoring toward losing weight, increasing strength, and for other health conditions, such as Parkinson's disease or back pain. Some attendees felt under-challenged as the exercises did not meet their needs: “*you get up to somewhere like 30* [repetitions] *doing something, well that is enough of doing it, you want to find either something else that is going to get you back down to about 5 or 10 to begin to build up*” (P595), while others enjoyed a less demanding approach “*you are not forced, you are encouraged*” (P205). HCPs did ask attendees their exercise needs at the start but observed “*a lot of them won't do that right at the start, that's what I've found, um but then I'll have sort of like over the time they'll sort of say*,” “*Oh you know I've been having problems with my knee,” or whatever, “is there anything I can be doing around my knee?”* (PT,C1).

#### Flexible Education and Discussion

A person-centred care approach was evident in how DCEP created a space for people to access education in an open and flexible way. A short educational talk and discussion were held at each session, covering varied health topics, for example, foot care, exercise, heart care, mental health, diet and nutrition, BP care, self-monitoring, mindfulness, and arthritis. The session topics were suggested by attendees, and the subsequent discussion was wide ranging. All HCPs could see the benefits of this attendee-driven discussion: “*you always get some people who've got some experience, who can help others, who can relate to their personal stuff* ” (Grief Intervention Counsellor,C2). Some HCPs were, however, concerned about delivering the education sessions, “*really facilitate rather than, I guess, preach at. Um, which was, to be fair, quite daunting at the start”* (Pharmacist,C2), and worried about what information attendees needed: “*don't know what they want to know until you tell them what they need to know*” (Grief Intervention Counsellor,C2). HCPs were also worried about being under-prepared for attendee questions: “*just questions coming my way where I might not know the answer*” (PT,C2), being unable to redirect informal discussions, overloading attendees with information, and the ability of attendees to retain information. Some HCPs recommended a structure of a short formal presentation that included key points about the topic, followed by a discussion session, while others felt it was important to be flexible: “*Anyway, you've got to have a little bit of an agenda to cover information that people need to know. Then you've also got to allow for flexibility”* (Grief Intervention Counsellor,C2). To help attendees retain information, some HCPs wanted to include additional written material in the form of a workbook, while other HCPs worried about the potential to exclude attendees with low levels of literacy.

This person-centred approach to the educational sessions motivated attendance at DCEP, “*And the thing I like about that type of programme is there an educational speech after each thing there, so, it keeps that momentum interest and that. And it's all surrounded around diabetes and all the… and that, before, you know, before I attended that programme, I couldn't control what was going in my mouth, in terms of eating and that, you know, fatty foods, all the wrong foods and that*” (P542), and were found to be informative: “*Because I didn't have any information before I came to this course and things throughout the time in the three months that you'll be learning, is was like the little light bulbs were going off* ” (P238). The way education sessions were presented was appreciated: “*a very, very good speaker, she put it down in ordinary terms and it was easy enough to follow”* (P280). There were opportunities to ask questions and share experiences: “*Sometimes you were just getting to the interesting stages and it was cut short because the next group was coming in and they were starting to disturb the flow of thinking*” (P205).

#### HCP Training

The core values of DCEP are truly person-centred and are made explicit in the HCP training. These core DCEP values are based on the spirit of motivational interviewing, described here by the DCEP developer:

*How can I work with you to be helpful? Non-judgemental, accepting, come from that place and that space because too many people don't necessarily have, unfortunately, healthcare experiences that they really enjoy. Particularly people with multiple, long-term health conditions. They see numerous healthcare professionals, numerous times, and are told, “do this, do this, and do this,” and not many people are particularly good at “doing this, doing this, doing this,” hence they keep on presenting with continuously deteriorating health concerns* (DCEP Developer).

Such an approach to patient care may have differed from what the HCPs had previously used in clinical practise and one which may require more training than we perhaps provided. The DCEP developer reflected that this approach could not be fully taught in a traditional training model, requiring concurrent working experience, deliberate practise and feedback from a trainer experienced in the approach. It was apparent that some HCPs needed more clarity about DCEP values and details: “*I didn't, um, get any formal discussion around what I was supposed to do. I just sort of, it was just a, flying on the seat of my pants really…because nothing was handed over.”* (Nurse,C2). It was suggested that a written package would be a useful resource for future consistency and clarity around programme values and HCP roles.

Some training was available to all HCPs; however, the extent of this training varied across HCPs for two primary reasons: staff turnover and if DCEP values differed or not from usual practise. DCEP delivery was reliant on accessing a regular team of a nurse and physiotherapist, plus a variety of educational speakers. Orientation and information sessions were available to the nurse and physiotherapy team; however, due to an unexpected high turnover of DCEP nurses, information about DCEP and the roles of nurses were not always clear. A nurse manager described some of the difficulties: “*And keeping the communication flowing when there's new staff, um, trying to orientate new staff members to the programme… I think, where, from my point of view, I probably needed to be a little bit more hands-on, with making sure that the staff were having a good orientation from the programme*” (Nurse Manager,C2).

### Theme 2: Attention to Logistics and Administration

This theme reflects what participants thought was important in the management and organisation of DCEP. It is a smaller theme than the previous one, as when DCEP attendees were asked about their experience of DCEP, all they focussed on was its person-centred aspects. The logistics and processes that enable DCEP to occur (for example, factors such as travel, exercise equipment, costs, physical space, location and time) were only talked about when specifically prompted to by the interviewer: “*Yes, well, before I went, before I started, I actually went to see where it was. And I found it, and it's good, yes*” (P933). Although some attendees experienced difficulties with some of these delivery factors, it appeared that these difficulties were overcome to access DCEP: “*we organise things so that, OK, those days we did whatever we had to do in town, you know, get groceries, and make other appointments and what have you*” (P280). Attendees not worrying about these factors may be because of the prior preparation undertaken by HCPs “*it has been dealt with really well and handled really well and delivered really well*” (P686).

For HCPs, some logistical factors were considered essential, such as choosing a venue with adequate location and physical space: “*accessible for, sometimes, older, frailer people with physical disabilities; good parking, ideally, free parking, trying to break down any barriers to access; and maybe a location closer to the high-needs communities where we wanted to work in, and big enough to house up to 25 people*” (DCEP Developer). Stream-lined administration was also considered essential: “*I think there's probably just more admin than you think… there is that liaison with the GP* [General Practitioners] *both after the groups and during if there's concerns. There's the ringing up of people that haven't turned up and spend some time calling them and sort of saying hey, you know, I hope you're ok, you know and try and encourage them back”* (PT,C1). Important though was that the administrator be well-trained to enable efficient processes, as one administrator told us how she was “*Thrown in, in the deep end.So I was just learning completely blind … so there was a lot of coordinating and making sure that we had all our ducks in a row basically, to keep it going*” (Long-term Health Administrator, C2).

Attendees and HCPs felt DCEP should be more accessible to working people with perhaps an evening programme or a more flexible approach: “*Or even condensing some of it, like having education sessions but outside of work time and once a month or something like that rather than every week”* (PT,C1).

## Discussion

The overarching aim of DCEP is to support adults living with T2D to take control of their health and to live well with their long-term condition, with a specific emphasis on exercise self-management and exercise self-efficacy to engage people with T2D with exercise for a long term. With this in mind, this study explored with DCEP attendees and involved HCPs what they considered the crux of DCEP to be and what makes people attend the initial 12-week intervention or not. The key to why people appeared to attend DCEP is evident in the two themes, namely, person-centred focus of DCEP and the attention given to the logistics of running DCEP and the importance of good administration. These aspects were important to attendees, although, as apparent in the findings, more consideration is required for some aspects to improve DCEP delivery, such as more training. In this discussion, we focussed on four important learning points arising from our themes, *person-centred care*, and its subthemes, and *attention to logistics and administration*. These insights may be informative to others wishing to develop similar programmes to DCEP. First, a person-centred care approach is important and it should, in the first instance, concentrate on the “person” *via* health status monitoring and education and social aspects of the programme, as these aspects appeared key to facilitating exercise engagement. Second, attendees valued the knowledgeable HCPs, people who they trusted and felt safe with. Third, having such HCPs was, however, not enough in itself, as to conduct DCEP in the person-centred way that was valued, these health professionals required specific training. The fourth insight is that while the known principles of running a community-based exercise class must be attended to, well-trained administrative support is also essential. Below, we discuss each point in more detail.

A prominent learning for us, within person-centred care, was that while the exercise component of DCEP initially motivated attendees to attend, the monitoring, educational, and social aspects that focussed on the “person” who sustained the engagement and the exercise component then appeared to be a “by-product” of attending. We had assumed that DCEP attendees would attend the exercise as they knew it would be good for managing their health and that the social side would facilitate this attendance. Whereas, DCEP attendees appeared to come for the monitoring and social aspects with the exercise as an added bonus. This insight, in which we, as Nicholls et al. (24) have eloquently described, thought of DCEP as “the medical deployment of therapeutic exercise” [24, p. 407] but participants considered it more as “see the value in exercise as expression and indulgence, luxury, and creativity” [24, p. 408], may be useful to those wishing to develop exercise programmes for people living with long-term conditions: namely, focus on being interested in the health and overall well-being of a person, and introduce exercise as an add-on. An appreciation of a focus on person-centred health was evident when DCEP attendees spoke of their wish to regularly check their BPs and BSLs and get individual advice, which was accommodated by the attending nurse. The nurses also valued this interaction as it gave them an opportunity to subtly monitor attendees. This regular “check-in” within DCEP may be critiqued for driving patient dependency on HCPs, with one nurse saying, “*It's sort of created this whole monster, really, of a line-up for blood pressures,”* but could be considered important in maintaining a person-centred approach to facilitate engagement. In terms of the physiotherapists, DCEP attendees liked how the exercises were individually tailored to their requirements, for example, a focus on exercise to prepare them for forthcoming surgery or to manage back pain. Further, attendees valued the education sessions, which were attendee-driven. The social, educational, and supervisory support provided by DCEP may emulate behaviour change techniques identified in systematic reviews as being associated with clinically significant reductions in glycated haemoglobin (25). Such techniques include “instruction on how to perform a behaviour,” “behavioural practise/rehearsal,” “action planning” and “demonstration of the behaviour” (11, 12). The ongoing, supervised nature of DCEP, along with the vicarious experiences attendees gained from each other, could be considered to represent these identified techniques.

Second, a key attraction of DCEP for attendees was the presence of knowledgeable nurses and physiotherapists. The attendees were aware that these HCPs indirectly monitored how they were doing and provided tailored advice and guidance; as a result, they felt safe exercising. Having two experienced, knowledgeable HCPs present may be considered an expensive option; however, this strategy enabled safe continued exercising, which may perhaps have prevented more serious deteriorations in health, and conversely, in the long run, be a more inexpensive approach than usual care. Furthermore, DCEP attendees said that they preferred DCEP over other exercise gyms in town because of these supportive relationships they had with knowledgeable staff and with other attendees and because it was cheaper. The need for supervisory exercise support has been evidenced by a recent network meta-analysis that found that a combined (aerobic and resistance) supervised exercise programme was more effective in glycaemic control than a non-supervised programme (10). While lay trained supervisors are helpful, they may not address all risk factors for T2D. For example, a UK-based community-based intervention for people with T2D, the “Living Well, Taking Control” programme, involved 4 weekly 2-h group sessions held in local community venues led by trained voluntary lay facilitators and had moderate effectiveness on weight-related outcomes but limited effect on physical activity engagement (26). The importance of having knowledgeable HCPs involved in community-based exercise programmes was stressed when people with balance and mobility limitations were consulted on the implementation of a bespoke movement and exercise programme (27). Such expert knowledge provided a sense of trust and reassurance that the programme was safe and appropriate (27).

The third key point is that, to run DCEP, all the HCPs involved require good training, including an emphasis on person-centred care. The DCEP spirit or “heart-set,” based on that of motivational interviewing (18), of partnership, acceptance and compassion, are subtle and yet appear to be the keys elements appreciated by attendees and facilitators of their attendance. The involved physiotherapists and nurses need to be trained in these principles, in how, as explained by the DCEP developer, “to be helpful,” as this approach may be different from how care and education is often didactically delivered. This is akin to what Rogers describes as “way of being” of a clinician with the person they are supporting (28). The HCP educators also needed experience and good training as the education in DCEP is delivered in a flexible manner, tailored to what attendees request, with high value given to participant-driven discussions. This tailored approach could be daunting for the HCP educators who were never sure what questions would be asked of them. As is often the case in health care systems, staff turnover can be high, so training is important and needs to be frequent and ongoing (27).

The fourth point is that although the practicalities of running DCEP (such as administration, venue, time of day, parking, access, location size, safety) are important (27, 29, 30), the DCEP attendees did not focus on these aspects of DCEP. This was perhaps because these factors had been attended to upfront and should be “a given” for any community-run exercise and education group. However, for the administrators, there was a lot of behind-the-scenes coordinating work, and training to do this was considered important. One observation made was that attendees would have preferred flexibility in terms of the time of day to attend. As previously argued, while these suggestions of administrative training and flexibility may increase the costs of delivery, they could enhance attendance and benefits, ultimately potentially decreasing overall health costs.

Given the above points, does DCEP achieve its aims of enhancing exercise self-management and exercise self-efficacy for those living with T2D? Self-management support has been defined as including “all the actions taken by people to recognise, treat, and manage their own health care independently of or in partnership with the health care system. People feel more confident and engaged when they are encouraged to self-manage by professionals, therefore supporting self-management is key to prioritising person-centred care” [(31), p. 3]. It could be argued that DCEP supports people in a person-centred care approach to self-manage by providing an environment conducive to motivation to exercise safely. This finding resonates with a recent meta-review, which reported that self-management programmes that were multifactorial and of longer delivery (over 10 h) were more effective than a short discrete intervention, emphasising the ongoing supportive nature required of such programmes (4). Additionally, given the vacillating nature of a long-term health condition such as T2D, a co-created flexible approach with appropriate cultural tailoring is considered optimal, highlighting the person-centred approach (4, 12, 32).

The strengths of this study are in the number, range and ethnicity of people (both DCEP attendees and DCEP personnel in two centres) interviewed, providing diversity in perspective, and the rich depth of data collected. Limitations include only interviewing those people who attended DCEP and not those who, in the larger RCT, attended the control group, or dropped out early from the DCEP classes, potentially biassing the perceptions held. Further, DCEP attendees were interviewed following attendance at the 12-week initial DCEP programme and thus do not represent those DCEP attendees that did or did not continue with DCEP in the continuing classes. We thus have elucidated what facilitates engagement in the 12-week initial programme, but not what motivates, or not, longer term engagement in the continuing maintenance exercise programme. Longitudinal research of the cost benefits of the entire DCEP programme is essential and is presently been undertaken as part of the current RCT.

DCEP did motivate people with T2D to engage in exercise, and attendance over the initial 12-week programme was good. Important to this engagement was the emphasis on a person-centred approach that focussed on the monitoring, educational and social aspects of the programme, which in turn facilitated exercise engagement. Imperative was knowledgeable HCPs who required training in the delivery of person-centred care, underpinned by the spirit of motivational interviewing, to tailor the exercise and education to the individual. Equally important are optimal exercise environments and well-trained administrative support. This study has reported on the tangible or concrete elements of DCEP constructed from the participant interviews, but underlying these tangible elements were many subtle nuances that also supported attendee engagement. These nuances were less obvious but likely equally important and we will report these in a subsequent article. Future research will need to focus on the elements of the second part of the DCEP programme, which is the ongoing twice-weekly maintenance exercise class, and if and why people continue to engage long term in exercise. This lifelong engagement is key for exercise to be able to facilitate adults living with T2D to take control of their health and to live well with their long-term condition.

## Data Availability Statement

The raw data supporting the conclusions of this article will be made available by the authors, without undue reservation.

## Ethics Statement

This study was reviewed and approved by the NZ Health and Disability Ethics Committee (17/CEN/241). Participants provided their written informed consent to participate in this study.

## Author Contributions

LH is the primary author and the principle investigator of the research project, wrote the introduction, methods, and discussion sections of the paper and edited the findings sections. CH was the lead clinical author of this paper and organised and supervised all the clinical staff involved in the study, developed DCEP, and contributed significantly to the data analysis and editing of the paper. DK was the lead project manager and organised all the interviews and facilitated a number of the interviews and contributed significantly to the data analysis and editing of the paper. CS led and did most of the qualitative analysis of this project and led the writing of the findings section of the paper and assisted in editing the paper overall. All authors contributed to the article and approved the submitted version.

## Conflict of Interest

The authors declare that the research was conducted in the absence of any commercial or financial relationships that could be construed as a potential conflict of interest.
